# Regular use of paracetamol and risk of liver cancer: a prospective cohort study

**DOI:** 10.1186/s12885-023-11767-5

**Published:** 2024-01-04

**Authors:** Liang Tian, Ningning Mi, Leiqing Wang, Chongfei Huang, Wenkang Fu, Mingzhen Bai, Long Gao, Haidong Ma, Chao Zhang, Yawen Lu, Jinyu Zhao, Xianzhuo Zhang, Ningzu Jiang, Yanyan Lin, Ping Yue, Bin Xia, Qiangsheng He, Jinqiu Yuan, Wenbo Meng

**Affiliations:** 1 The First Clinical Medical College, Lanzhou University, Lanzhou, Gansu, China; 2 Department of General Surgery, The First Hospital of Lanzhou University, Lanzhou, Gansu, 730000 China; 3 Clinical Research Center, Big Data Center, The Seventh Affiliated Hospital, Sun Yat-Sen University, Shenzhen, Guangdong, 518107 China

**Keywords:** Liver cancer, Paracetamol, Prospective cohort study, UK Biobank

## Abstract

**Background:**

Paracetamol induces hepatotoxicity and subsequent liver injury, which may increase the risk of liver cancer, but epidemiological evidence remains unclear. We conducted this study to evaluate the association between paracetamol use and the risk of liver cancer.

**Methods:**

This prospective study included 464,244 participants free of cancer diagnosis from the UK Biobank. Incident liver cancer was identified through linkage to cancer and death registries and the National Health Service Central Register using the International Classification of Diseases (ICD)-10 codes (C22). An overlap-weighted Cox proportional hazards model was utilized to calculate the hazard ratio (HR) and 95% confidence interval (CI) for the risk of liver cancer associated with paracetamol use. The number needed to harm (NNH) was calculated at 10 years of follow-up.

**Results:**

During a median of 12.6 years of follow-up, 627 cases of liver cancer were identified. Paracetamol users had a 28% higher risk of liver cancer than nonusers (HR 1.28, 95% CI 1.06–1.54). This association was robust in several sensitivity analyses and subgroup analyses, and the quantitative bias analysis indicated that the result remains sturdy to unmeasured confounding factors (E-value 1.88, lower 95% CI 1.31). The NNH was 1106.4 at the 10 years of follow-up.

**Conclusion:**

The regular use of paracetamol was associated with a higher risk of liver cancer. Physicians should be cautious when prescribing paracetamol, and it is recommended to assess the potential risk of liver cancer to personalize the use of paracetamol.

**Supplementary Information:**

The online version contains supplementary material available at 10.1186/s12885-023-11767-5.

## Introduction

Liver cancer rank as the sixth most common cancer and was the third leading cause of global cancer-related deaths in 2020, with approximately 906,000 incident cases and 830,000 deaths [1, 2]. The major risk factors for liver cancer include chronic infection with hepatitis B virus (HBV) or hepatitis C virus (HCV), consumption of food contaminated with aflatoxins, smoking, excessive alcohol consumption, obesity, type 2 diabetes, and certain medication use.

Paracetamol (acetaminophen) is widely used for fever reduction and pain relief in general situations [3], but its hepatotoxicity is a primary factor contributing to drug-induced liver failure [4]. Several animal studies have demonstrated that drug-induced liver failure is related to the hepatocarcinogenicity of paracetamol [5, 6]. In line with the findings of animal studies, many population-based studies have reported that high-dose acetaminophen may also cause liver injury [7, 8]. Previous epidemiological studies have also investigated the association between paracetamol and liver cancer risk. A population-based study based on Danish registries found that paracetamol users have a nonsignificantly higher risk of liver cancer compared to the general Danish population [9]. A nested case‒control study based on the UK’s Clinical Practice Research Datalink revealed that paracetamol was associated with a slightly increased risk of liver cancer (OR = 1.18, 95% CI = 1.00–1.39) [10]. Both studies are thought-provoking, but their validity is limited by the absence of accounting for crucial covariates.

Given the widespread use of paracetamol and the public health threat of liver cancer, further evaluation of the effects of paracetamol on liver cancer is warranted. Thus, we conducted this prospective analysis of the UK Biobank cohort to investigate the associations between paracetamol use and subsequent risk of liver cancer.

## Materials and methods

### Study and participants

The United Kingdom Biobank is a large-scale, prospective, population-based cohort of over 500,000 individuals aged 37–73 years who were recruited from 21 assessment centers across the U.K. in 2006–2010. All eligible participants were invited to complete touchscreen questionnaires, face-to-face interviews, physical measurements, and biological sample collection. Detailed information about the project is available on the website (https://www.ukbiobank.ac.uk/) and in previous studies [11]. The UK Biobank was approved by the North West Multi-Center Research Ethics Committee. All participants provided written informed consent prior to data collection. In this study, we excluded 36,865 participants with a diagnosis of cancer and 1,301 participants who had missing data on other covariates. The final analyses included 464,244 participants.

### Exposure assessment

At baseline, regular use of paracetamol was first evaluated by participants using a touchscreen questionnaire and subsequently confirmed by a UK-biobank trained staff. “Regular use” was defined as taking the medication in most days of the week for the last 4 weeks. Information regarding the doses and duration of paracetamol use was not collected.

### Ascertainment of outcome

Incident liver cancer cases within the UK Biobank cohort were identified by ICD-10 codes C22. This information was recorded from cancer and death registries from the Health and Social Care Information Centre (in England and Wales) and the National Health Service Central Register (in Scotland). The diagnosis of liver cancer was confirmed by medical records, pathology reports, imaging results, and death certificates. Details of the methods can be found on the UK Biobank website.

### Covariates

Covariates were determined from a touchscreen questionnaire and verbal interview at baseline. These included sociodemographic factors (age, sex, and race) and lifestyle factors (smoking, alcohol consumption, sleep time, and diet habits). The index of multiple deprivations based on the postcode of residence was determined as a composite measure of socioeconomic status. Physical activity was estimated by the validated Short International Physical Activity Questionnaire (IPAQ) for all individuals. Comorbidities (hypertension, diabetes, hyperlipidemia, viral hepatitis, cirrhosis) and medication use (multivitamins, mineral supplements, aspirin, ibuprofen, NSAIDs, PPIs, histamine-2 receptor antagonists, antihypertensive drugs, antidiabetic drugs, and statins) were assessed based on self-reported medical history. Body mass index (BMI) was calculated by dividing weight by the square of height (kg/m2).

### Statistical analysis

Person-years were calculated from the date of return of the baseline questionnaire to the date of first diagnosis of liver cancer, death, or the end of follow-up (31 December 2020), whichever came first. Cox proportional hazards models with age as the timescale were fitted to calculate the hazard ratios (HRs) with 95% confidence intervals (CIs).

We employed an overlap propensity score weighting approach to address potential confounding. First, we used multivariate logistic regression model conditioned on baseline covariates, including age, sex, centers, race, socioeconomic status (index of multiple deprivation), smoking status, alcohol consumption, physical activity, fruit and vegetable intake, meat intake, sleep time, BMI, concomitant comorbidities (hypertension, diabetes, hyperlipidemia, viral hepatitis, cirrhosis), current medication (multivitamins, mineral supplements, aspirin, ibuprofen, NSAIDs, PPIs, histamine-2 receptor antagonists, antihypertensive drugs, antidiabetic drugs, and statins), overall health rating, long-standing illness, and family history of cancers, to calculate the propensity score for paracetamol use. The overlap weight based on the propensity scores was then applied to establish a pseudo population in which the measured confounders were balanced between paracetamol users andnonusers. Standardized mean differences (SMDs) were calculated before and after weighting to assess covariate balance, with SMD less than 0.1 considered negligible [12]. A weighted Kaplan–Meier curve was generated to characterize the cumulative incidence of liver cancer over time. Weighted Cox regression models were used to estimate marginal HRs with 95% CIs for the effect of paracetamol use on liver cancer risk. Schoenfeld’s tests were employed to check the proportional hazard assumption and no violation was detected. To present the association easily, we calculated the number needed to harm (NNH) with the method described by Altman and Andersen [13].

To further investigate potential effect modifiers, we conducted subgroup analyses stratified by sex, age, obesity, smoking and drinking status. We performed several sensitivity analyses to test the robustness of our findings. First, we performed a lagged analysis of the exposure for 2 years to minimize the potential for protopathic bias. Second, we excluded participants with viral hepatitis and cirrhosis to control the potential impact of health conditions. Third, we employed an alternative inverse probability treatment weighting (IPTW) approach to further mitigate the impact of confounding variables. The IPTW method aimed to achieve a balanced distribution of measured confounders between paracetamol users and nonusers. The propensity scores for paracetamol use were derived from a multivariable logistic regression model. We evaluated the balance of covariates between the two groups by computing the standardized mean differences (SMDs) before and after weighting. Covariates with SMDs less than 0.1 were considered negligibly unbalanced. Weighted Cox regression models were used to estimate the HRs and 95% CIs as described by Austin et al. [14]. Last, we calculated the E-value to estimate the potential role of unmeasured confounders, which represents the minimum strengths of association between an unmeasured confounder and exposure or outcome that can fully explain away a specific treatment–outcome association, conditional on the measured covariates [15]. All analyses were conducted using R software (version 4.1.0, R Foundation for Statistical Computing, Vienna, Austria).

## Results

This study included 464,244 participants from the UK Biobank, of which 103,018 (22.19%) participants reported regular use of paracetamol. At baseline, regular paracetamol users were more likely to be less physically active, consume less alcohol, intake less vitamin and mineral supplements, and have a higher rate of longstanding illness. Paracetamol users also had a higher rate of taking other medications (such as ibuprofen, PPIs, and H2RAs). After weighting, all covariates were well-balanced (SMDs below 0.10, Table 1).

**Table 1:** Baseline characteristics of participants by paracetamol use before and after weighting

	**Before weighting**			**After weighting**^**a**^		
	**Non paracetamol user**	**Paracetamol user**	**SMD**	**Non paracetamol user**	**Paracetamol user**	**SMD**
**N**	361,226	103,018		71,398.71	71,398.71	
**Mean (SD) age, years**	56.99 (8.04)	56.03 (8.30)	0.118	56.48 (8.15)	56.36 (8.27)	0.014
**Male, N (%)**	178,773 (49.5)	36,695 (35.6)	0.283	27,623.2 (38.7)	27,623.2 (38.7)	< 0.001
**White, N (%)**	342,623 (94.9)	95,880 (93.1)	0.075	66,900.8 (93.7)	66,900.8 (93.7)	< 0.001
**Smoking status, N (%)**			0.069			< 0.001
Current	36,650 (10.1)	12,422 (12.1)		8258.7 (11.6)	8258.7 (11.6)	
Previous	122,436 (33.9)	35,717 (34.7)		24,751.2 (34.7)	24,751.2 (34.7)	
Never	202,140 (56.0)	54,879 (53.3)		38,388.8 (53.8)	38,388.8 (53.8)	
**Alcohol consumption, N (%)**			0.213			< 0.001
Daily or almost daily	77,155 (21.4)	16,871 (16.4)		12,511.3 (17.5)	12,511.3 (17.5)	
1–4 times a week	181,284 (50.2)	47,300 (45.9)		33,680.3 (47.2)	33,680.3 (47.2)	
One to three times a month	38,481 (10.7)	13,112 (12.7)		8706.7 (12.2)	8706.7 (12.2)	
Special occasions only/Never	64,306 (17.8)	25,735 (25.0)		16,500.4 (23.1)	16,500.4 (23.1)	
**Physical activity, N (%)**			0.125			< 0.001
Low	52,607 (14.6)	17,572 (17.1)		11,659.5 (16.3)	11,659.5 (16.3)	
Moderate	118,653 (32.8)	32,815 (31.9)		23,012.7 (32.2)	23,012.7 (32.2)	
High	120,927 (33.5)	29,774 (28.9)		21,374.4 (29.9)	21,374.4 (29.9)	
**Fruit and vegetable intake ≥ 5 portions per day, N (%)**	136,334 (37.7)	37,978 (36.9)	0.018	26,427.3 (37.0)	26,427.3 (37.0)	< 0.001
**Red and process meat intake, times per day, N (%)**			0.021			< 0.001
< 2 times per day	54,505 (15.1)	15,474 (15.0)		10,805.8 (15.1)	10,805.8 (15.1)	
2–3 times per day	104,716 (29.0)	28,974 (28.1)		20,240.2 (28.3)	20,240.2 (28.3)	
3–4 times per day	54,733 (15.2)	15,703 (15.2)		10,914.2 (15.3)	10,914.2 (15.3)	
> 4 times per day	147,272 (40.8)	42,867 (41.6)		29,438.5 (41.2)	29,438.5 (41.2)	
**Mean (SD) sleep time, hours**	8.15 (1.12)	8.06 (1.23)	0.075	8.09 (1.19)	8.09 (1.20)	< 0.001
**Vitamin, N (%)**	50,416 (14.0)	18,438 (17.9)	0.108	11,983.3 (16.8)	11,983.3 (16.8)	< 0.001
**Mineral, N (%)**	73,361 (20.3)	25,283 (24.5)	0.102	16,652.0 (23.3)	16,652.0 (23.3)	< 0.001
**Mean (SD) body mass index**	27.24 (4.64)	28.07 (5.17)	0.168	27.83 (5.12)	27.83 (4.98)	< 0.001
**Health rating, N (%)**			0.371			< 0.001
Poor	67,545 (18.7)	10,337 (10.0)		8239.8 (11.5)	8239.8 (11.5)	
Fair	213,278 (59.0)	55,398 (53.8)		40,048.2 (56.1)	40,048.2 (56.1)	
Good	68,861 (19.1)	29,159 (28.3)		18,678.2 (26.2)	18,678.2 (26.2)	
Excellent	11,542 (3.2)	8124 (7.9)		4432.5 (6.2)	4432.5 (6.2)	
**Long-standing illness, N (%)**	101,782 (28.2)	40,299 (39.1)	0.241	25,925.4 (36.3)	25,925.4 (36.3)	< 0.001
**Cancer history, N (%)**	125,244 (34.7)	36,327 (35.3)	0.012	25,092.3 (35.1)	25,092.3 (35.1)	< 0.001
**Hyperlipidemia, N (%)**	66,715 (18.5)	18,916 (18.4)	0.003	13,306.4 (18.6)	13,306.4 (18.6)	< 0.001
**Viral hepatitis, N (%)**	803 (0.2)	212 (0.2)	0.004	146.0 (0.2)	146.0 (0.2)	< 0.001
**Cirrhosis, N (%)**	327 (0.1)	137 (0.1)	0.013	86.7 (0.1)	86.7 (0.1)	< 0.001
**Hypertension, N (%)**	211,875 (58.7)	59,036 (57.3)	0.027	41,291.1 (57.8)	41,291.1 (57.8)	< 0.001
**Diabetes, N (%)**	18,662 (5.2)	5389 (5.2)	0.003	3776.7 (5.3)	3776.7 (5.3)	< 0.001
**Aspirin, N (%)**	50,662 (14.0)	15,396 (14.9)	0.026	10,445.4 (14.6)	10,445.4 (14.6)	< 0.001
**Ibuprofen, N (%)**	37,672 (10.4)	33,205 (32.2)	0.552	17,118.0 (24.0)	17,118.0 (24.0)	< 0.001
**PPI, N (%)**	30,677 (8.5)	15,031 (14.6)	0.192	9217.7 (12.9)	9217.7 (12.9)	< 0.001
**H2RA, N (%)**	5899 (1.6)	3711 (3.6)	0.124	2058.5 (2.9)	2058.5 (2.9)	< 0.001
**Antihypertensive drugs, N (%)**	72,613 (20.1%)	21,647 (21.0%)	0.023	15,046.8 (21.1)	15,046.8 (21.1)	< 0.001
**Antidiabetic drugs, N (%)**	13,666 (3.8)	3755 (3.6)	0.007	2678.9 (3.8)	2678.9 (3.8)	< 0.001
**Statin, N (%)**	58,761 (16.3)	15,935 (15.5)	0.022	11,376.1 (15.9)	11,376.1 (15.9)	< 0.001

^a^Pseudo population created by applying overlap propensity score weighting approach. *PPI* proton pump inhibitor, *H2RAs* Histamine-2 receptor antagonists, *SMD* standardized mean differences

**Fig. 1:**
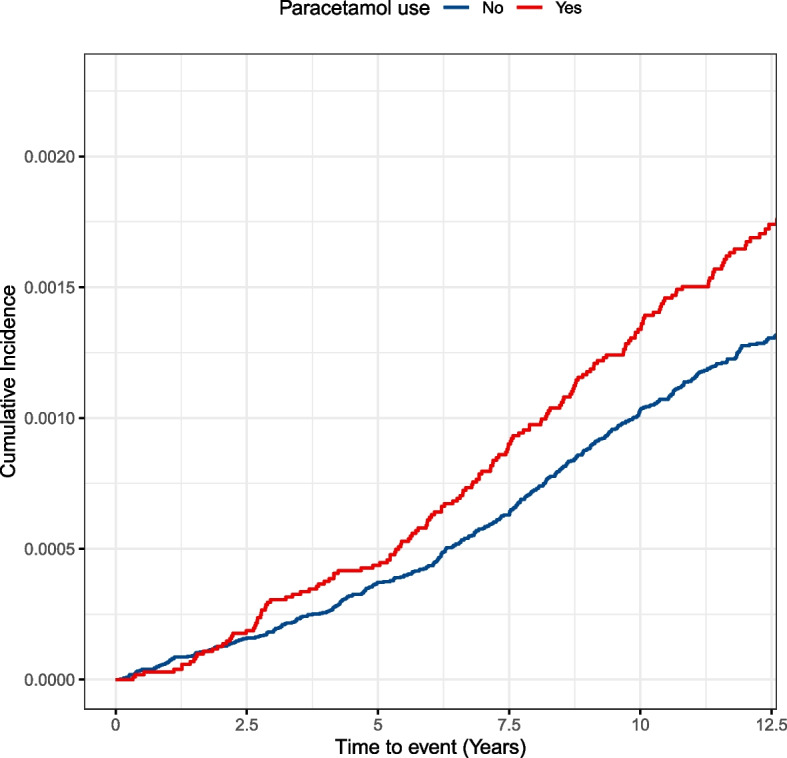
Cumulative incidence of liver cancer about paracetamol users and non-users in the overlap propensity score-weighted populations. The overlap weight adjusted Kaplan–Meier curves were generated based on the propensity score, and propensity score was derived by multivariate logistic regression conditional on aforementioned covariates in Table 2. The log-rank test was used to compare the survival difference between paracetamol users and non-users

Over a median follow-up of 12.6 years, we identified 171 cases of liver cancer among the 103,018 paracetamol users and 456 cases of liver cancer among 361,226 nonusers. In the crude model, regular paracetamol use was associated with a 41% increased risk of liver cancer compared with nonusers (HR 1.41, 95% CI 1.18–1.68). The association was attenuated after adjustment for potential confounders, but remained significan(HR 1.22, 95% CI 1.01–1.48). The overlap propensity score-weighted analysis showed a similar result (HR 1.28, 95% CI 1.06–1.54) (Table 2). The overlap weight-adjusted Kaplan–Meier curves demonstrated a higher cumulative incidence of liver cancer among paracetamol users compared to nonusers (Fig. 1). For straightforward interpretation of the effect, we calculated NNHs based on the weighted HR and the liver cancer incidence among non-paracetamol users. Every 10,227 (95% CI, 9506.5–22,336.8), 2147.6 (95% CI, 1920.4–5185.8), and 1106.4 (95% CI, 967.8–2800.7) paracetamol users may result in one case of liver cancer over 1, 5, and 10 years, respectively.The association between paracetamol and the occurrence of liver cancer became stronger as the duration increased, suggesting that the causality of our study is plausible. (Supplementary Figure S1).

**Table 2:** The association between paracetamol use and risk of liver cancer

	Case/Person-years	Hazard Ratio [95% Confidence Interval]		
		Crude model	Multivariable-adjusted model^a^	Propensity score-weighted model^b^
Non paracetamol user	456/4 304 030	1.00 [Reference]	1.00 [Reference]	1.00 [Reference]
Paracetamol user	171/1 229 448	1.41 [1.18, 1.68]	1.22 [1.01, 1.48]	1.28 [1.06, 1.54]

^a^Multivariable adjusted model: adjusted for age, sex, UK Biobank assessment centers, race, smoking, alcohol consumption, physical activity, fruit and vegetable intake, meat intake, sleep time, BMI, concomitant comorbidities (hypertension, diabetes, hyperlipidemia, viral hepatitis, cirrhosis), current medication (multivitamin, mineral supplements, aspirin, ibuprofen, PPI, histamine-2 receptor antagonists, antihypertensive drugs, antidiabetic drugs, and statin), overall health rating, Long-standing illness, and family history of cancers

^b^Propensity score–weighted model: propensity score was derived by multivariate logistic regression conditional on aforementioned covariates, and stabilized weight was calculated for each individual

**Fig. 2:**
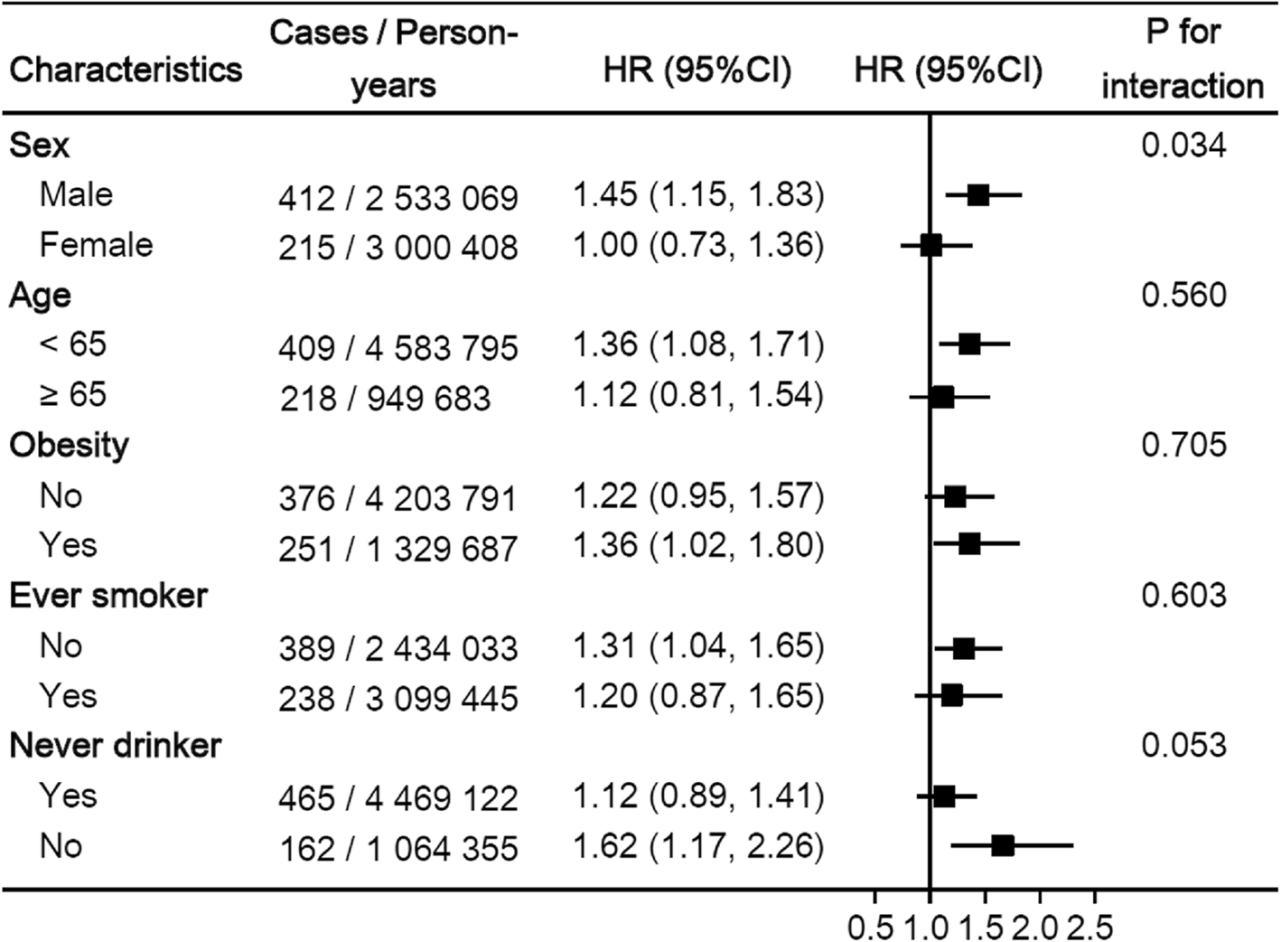
Subgroup analyses of regular use of paracetamol and risk of liver cancer. HR, hazard ratio; CI, confidence interval. Estimated effects were based on the propensity score-weighted model (see the footnote in Table 2)

Subgroup analyses showed that the associations between paracetamol use and the risk of liver cancer did not differ by age, obesity, smoking and drinking status, but a stronger positive association between paracetamol use and the risk of liver cancer was found among males (P-interactions = 0.034) (Fig. 2). In several sensitivity analyses, we observed no major changes in the associations between paracetamol use and the risk of liver cancer after lagging the exposure 2 years (HR 1.31, 95% CI 1.08–1.59), excluding the participants with viral hepatitis and cirrhosis (HR 1.25, 95% CI 1.03–1.52), using the stabilized inverse probability of treatment weighting analysis (HR 1.32, 95% CIs 1.08–1.60) (Table 3). In the estimate of the influence of unmeasured confounders, the E-value for the primary findings was 1.88, and the lower 95% confidence limit for the E-value was 1.31 (Figure S2).

**Table 3:** Sensitivity analysis for the association between paracetamol use and risk of liver cancer

	Case/Person-years	Hazard Ratio [95% Confidence Interval]		
		Crude model	Multivariable-adjusted model^a^	Propensity score-weighted model ^b^
**Lagging the exposure for 2 years**				
Non paracetamol user	411/4 297 789	1.00 [Reference]	1.00 [Reference]	1.00 [Reference]
Paracetamol user	158/1 227 698	1.46 [1.21, 1.75]	1.24 [1.02, 1.51]	1.31 [1.08, 1.59]
**Stabilized inverse probability of treatment weighting analysis**				
Non paracetamol user	456/4 304 030	1.00 [Reference]	1.00 [Reference]	1.00 [Reference]
Paracetamol user	171/1 229 448	1.41 [1.18, 1.68]	1.22 [1.01, 1.48]	1.32 [1.08, 1.60]
**After excluding viral hepatitis and cirrhosis**				
Non Paracetamol user	430/4 290 920	1.00 [Reference]	1.00 [Reference]	1.00 [Reference]
Paracetamol user	156/1 225 665	1.37 [1.14, 1.64]	1.20 [0.99, 1.47]	1.25 [1.03, 1.52]

^a^Multivariable adjusted model: adjusted for age, sex, UK Biobank assessment centers,race, smoking, alcohol consumption, physical activity, fruit and vegetable intake, meat intake, sleep time, BMI, concomitant comorbidities (hypertension, diabetes, hyperlipidemia, viral hepatitis, cirrhosis), current medication (multivitamin, mineral supplements, aspirin, ibuprofen, PPI, histamine-2 receptor antagonists, antihypertensive drugs, antidiabetic drugs, and statin), overall health rating, Long-standing illness, and family history of cancers

^b^Propensity score–weighted model using inverse probability weighting method: propensity score was derived by multivariate logistic regression conditional on aforementioned covariates, and stabilized weight was calculated for each individual

## Discussion

In this prospective cohort study involving over 460,000 participants, we found that regular paracetamol use was associated with a 28% increased risk of liver cancer after adjusting for potential confounding factors. Despite conducting subgroup analyses and several sensitivity analyses, the relationship between paracetamol use and liver cancer has persisted. Limited epidemiological studies have assessed the relationship between paracetamol usage and the risk of liver cancer. In 2002, based on a Danish cohort, Friss et al. reported a statistically nonsignificant elevation in the risk of liver cancer among paracetamol users (standardized incidence ratio 1.5, 95% CI 0.96–2.2) [9]. However, besides the influence of crucial covariates, the lack of information regarding the reasons for paracetamol use may also impact the study's validity, and this issue was also present in our study. In 2016, a nested case‒control study based on the UK’s Clinical Practice Research Datalink showed that paracetamol use was associated with a slightly increased risk of liver cancer (OR 1.18, 95% CI 1.00–1.39) [10]. This study used prospective data and included a total of 1195 cases of liver cancer, significantly enhancing the reliability of the results. These findings also strongly supported our study. In this study, we utilized the UK Biobank database and employed the overlap weighting approach to comprehensively adjust for confounding factors. The final result demonstrated a statistical association between paracetamol use and an increased risk of liver cancer.

The mechanism underlying the association between paracetamol use and liver cancer remains unclear, and is potentially attributed to hepatotoxicity. Paracetamol overdose results in the production of the toxic metabolite N-acetyl-p-benzoquinone imine (NAPQI), which depletes the antioxidant glutathione (GSH) and exacerbates oxidative stress via reactive oxygen species (ROS) generation. Ultimately, this cascade culminates in hepatic necrosis and cancerization [16, 17]. Furthermore, paracetamol has been found to potentially enhance the cleavage of β2-spectrin by caspase-3/7. These cleaved fragments could contribute to paracetamol-induced liver damage by influencing apoptosis and transcriptional processes, which are also linked to the potential development of liver cancer [18]. However, it is worth noting that various studies have suggested that therapeutic doses of paracetamol might exhibit antitumor effects on hepatoma [19]. For instance, paracetamol has been demonstrated to induce apoptosis in common hepatoma cell lines, such as HuH7 and SK-Hep1 cells [20]. Additionally, a study employing a quantitative systems toxicology (QST) model indicated that paracetamol does not pose a carcinogenic risk to humans at any dose [21]. Further research is needed to explore the underlying mechanisms involved.

Our study also indicated that male users of paracetamol had a higher risk of liver cancer, possibly attributed to gender-specific variations in the metabolism and clearance rate of paracetamol. Isaac et al. found that male mice were more sensitive to the toxicity of paracetamol, primarily due to a greater likelihood of paracetamol forming adducts with peroxiredoxin-6 and accelerated GSH depletion in male mice [22]. This finding supports our hypothesis. Additionally, differences in hormone levels between males and females may be a key factor [23]. However, the specific mechanisms are still unclear.

The primary strength of our study lies in its use of well-established prospective cohorts characterized by large sample sizes, extended follow-up durations, and thorough data collection encompassing lifestyle factors, medication use, and health conditions. This extensive dataset provided a strong foundation for effectively mitigating potential confounding effects. Moreover, the incorporation of multiple sensitivity analyses and subgroup analyses further enhanced the credibility and reliability of our findings.

This study has several limitations. First, in the UK Biobank, data on the indications for paracetamol usage were not collected, and specific details such as formulation, dosage, frequency, and duration of paracetamol administration were not documented. This limitation impeded our further analysis of those factors and may introduce potential bias. Second, the information on paracetamol use was collected only once at baseline and through self-reporting, which potentially impacted the reliability of the results and prevented us from assessing how changes in covariates and exposures over time might affect the risk of liver cancer.Third, paracetamol may be used to manage mild-to-moderate pain during the initial stages of liver cancer prior to diagnosis, which may lead to the emergence of reverse causation and amplify the risk of liver cancer associated with paracetamol use. The observational design of our study also limits the determination of causation. We lagged the exposure of paracetamol for 2 years to minimize the impact of potential reverse causation, and the results are still robust. Last, owing to the nature of observational studies, we must acknowledge the potential residual confounding effects of other unknown or unmeasured factors; therefore, further epidemiological and mechanistic studies are also necessary to address these limitations.

## Conclusion

Our study found that the regular use of paracetamol was associated with a higher risk of liver cancer. As this is an observational study, we cannot definitively establish a causal relationship; therefore, the findings should be interpreted with caution. Nevertheless, considering the widespread utilization of paracetamol and the potential threat of liver cancer to public health, this issue still warrants further attention. Furthermore, additional research is imperative to validate this association and elucidate the underlying mechanisms.

### Supplementary Information

**Additional file 1:** **Figure S1.** The required estimated number needed to harm for regular paracetamol users event of one case of liver cancer emerging. Compared to the control group, exposure to paracetamol resulted in 1 additional case of liver cancer per 10,000 users in the first year, and over time, 1 additional case of liver cancer per 1106 users at the10th year. **Figure S2.** E-value demonstrating required strength of unmeasured confounder to explain observed association between paracetamol use and liver cancer risk.

## Data Availability

This work has been conducted using the UK Biobank Resource. The UK Biobank is an open access resource by registering and applying at http://ukbiobank.ac.uk//register-apply/. Further information is available from the corresponding author upon request.

## Abbreviations

HR: Hazard ratios
CI: Confidence interval
IPTW: Inverse probability of treatment weighting
OR: Odds Ratio
