# Supplementary material for: Regular use of paracetamol and risk of liver cancer: a prospective cohort study
Source: BMC Cancer. 2024 Jan 4;24:33. doi: 10.1186/s12885-023-11767-5 (PMC10765829; doi:10.1186/s12885-023-11767-5)
Supplement: Supplementary file 1 — Additional file 1: Figure S1. The required estimated number needed to harm for regular paracetamol users event of one case of liver cancer emerging. Compared to the control group, exposure to paracetamol resulted in 1 additional case of liver cancer per 10,000 users in the first year, and over time, 1 additional case of liver cancer per 1106 users at the10th year. Figure S2. E-value demonstrating required strength of unmeasured confounder to explain observed association between paracetamol use and liver cancer risk. [file 12885_2023_11767_MOESM1_ESM.docx]

**Figure S1**

Figure S1. The required estimated number needed to harm for regular paracetamol users event of one case of liver cancer emerging. Compared to the control group, exposure to paracetamol resulted in 1 additional case of liver cancer per 10,000 users in the first year, and over time, 1 additional case of liver cancer per 1106 users at the10th year.

**Figure S2**


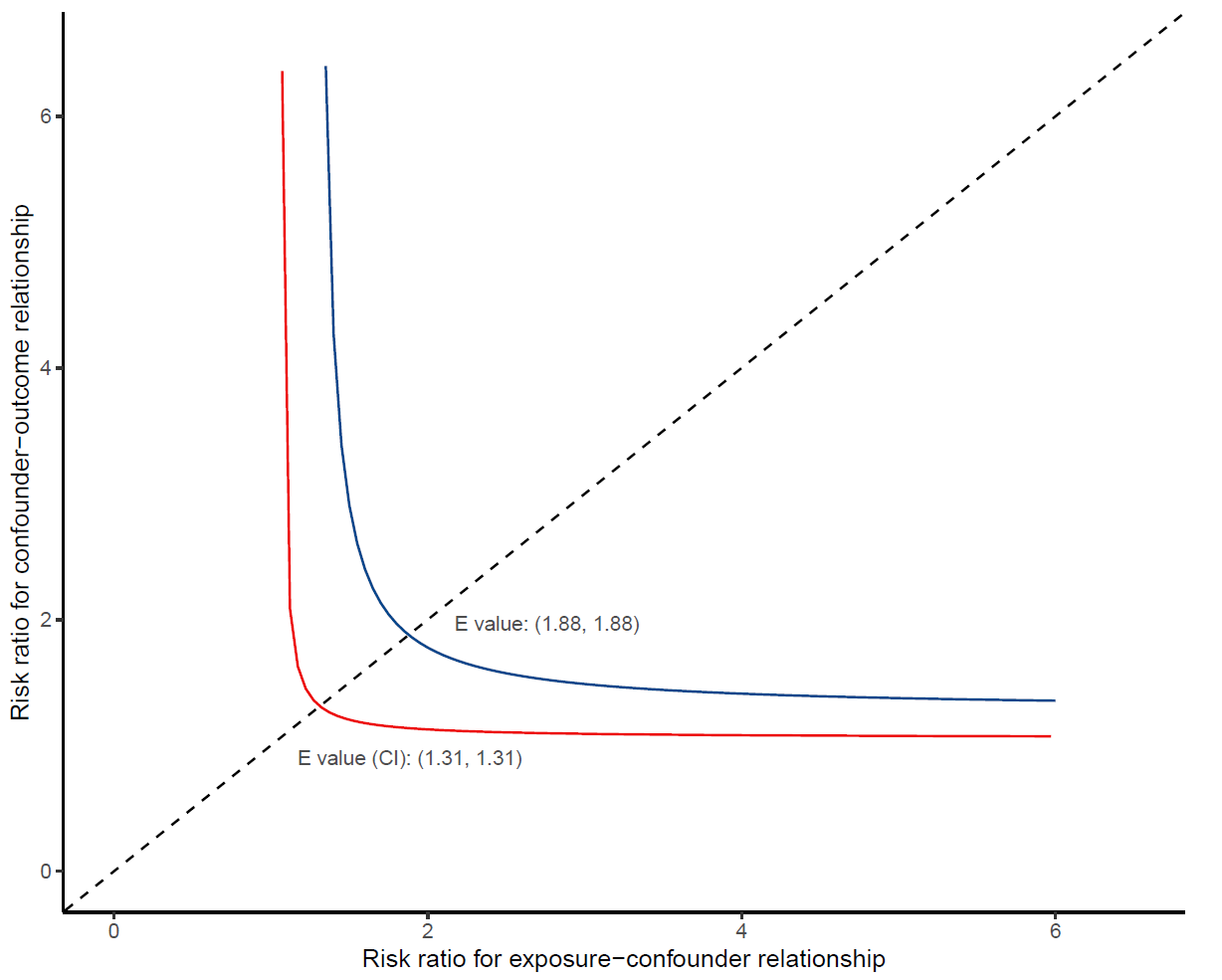


Figure S2. E-value demonstrating required strength of unmeasured confounder to explain observed association between paracetamol use and liver cancer risk.
